# Examining Potential Active Tempering of Adhesive Curing by Marine Mussels

**DOI:** 10.3390/biomimetics2030016

**Published:** 2017-08-21

**Authors:** Natalie A. Hamada, Victor A. Roman, Steven M. Howell, Jonathan J. Wilker

**Affiliations:** 1 Department of Chemistry, Purdue University, 560 Oval Drive, West Lafayette, IN 47907-2084, USA; nhamada@purdue.edu (N.A.H.); roman6@purdue.edu (V.A.R.); showell2@niu.edu (S.M.H.); 2 School of Materials Engineering, Purdue University, 701 West Stadium Avenue, West Lafayette, IN 47907-2045, USA

**Keywords:** adhesion, adhesive, byssus, catechol, DOPA, mussel, plaque, thread

## Abstract

Mussels generate adhesives for staying in place when faced with waves and turbulence of the intertidal zone. Their byssal attachment assembly consists of adhesive plaques connected to the animal by threads. We have noticed that, every now and then, the animals tug on their plaque and threads. This observation had us wondering if the mussels temper or otherwise control catechol chemistry within the byssus in order to manage mechanical properties of the materials. Here, we carried out a study in which the adhesion properties of mussel plaques were compared when left attached to the animals versus detached and exposed only to an aquarium environment. For the most part, detachment from the animal had almost no influence on the mechanical properties on low-energy surfaces. There was a slight, yet significant difference observed with attached versus detached adhesive properties on high energy surfaces. There were significant differences in the area of adhesive deposited by the mussels on a low- versus a high-energy surface. Mussel adhesive plaques appear to be unlike, for example, spider silk, for which pulling on the material is needed for assembly of proteinaceous fibers to manage properties.

## 1. Introduction

### 1.1 Catechols in the Sea

Mussels, sandcastle worms, and tube worms may be the most famous proponents of catechol chemistry [1]. These animals attach themselves to rocks using protein-based adhesives containing 3,4-dihydroxyphenylalanine (DOPA), for which the amino acid sidechain is a pendant catechol group. The surface adhesive properties of DOPA groups arise when the catechol ring is in the reduced (i.e., not oxidized) state [2,3]. Although some evidence does exist for oxidation when bonding at organic surfaces [4]. Cohesive strength for the glues is derived from one electron (to semiquinone) or two-electron (to quinone) oxidation of DOPA to then generate covalent cross-links, often with iron beginning such reactivity [5,6].

As our understanding of these natural systems has expanded, so too has there been a blossoming of biomimetic systems [7,8,9,10]. In a typical scenario, synthetic polymer backbones are synthesized to substitute for the protein matrix [11,12]. Derivatives of catechol are then appended to the polymer chain. In doing so, these efforts have given rise to new functional materials, including hydrogels, coatings, and adhesives. With greater understanding of the animals’ biology, chemistry, and mechanics will come the design ideas for new biomimetic systems.

### 1.2 Animals Managing Their Glue with Mechanical Forces

**Figure 1:**
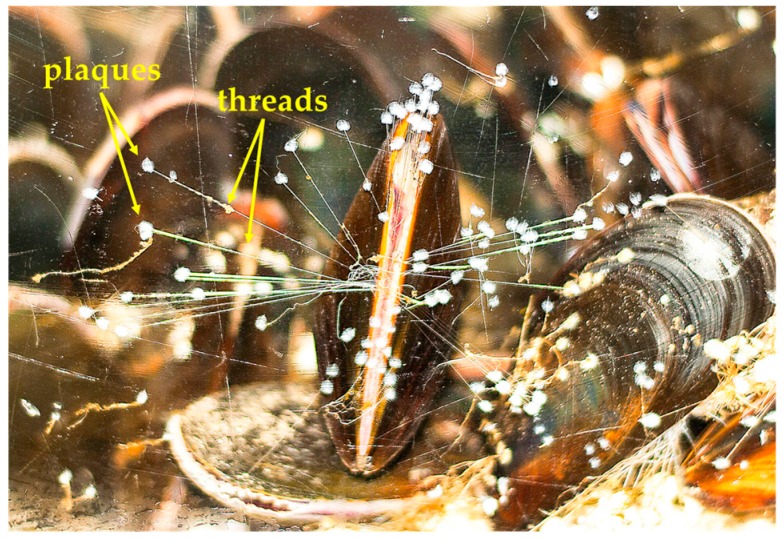
Marine mussel adhered to a glass wall of an aquarium tank. The byssal adhesive assemblies consist of adhesive plaques and threads which remain tethered to the byssal retractor muscles inside the shells.

Such a chemical perspective is helpful for materials design, although beyond the grasp of mussels, themselves. We have been wondering how the animals manage their adhesive. They are well known to attach atop substrates with the byssal plaque and thread structure visible in Figure 1. Our research group has been working with these shellfish for several years now [13,14,15,16]. During these studies, we have noticed that the animals are not completely passive after deposition of adhesive plaques. Mussels do not apply the glue and then simply sit around. Rather, upon closer observation, it appears as if the animals are pulling on their threads. This byssal tugging can be observed with movement of the animal while the threads remain relatively stationary, appearing as if the animals are shaking around a bit. Such motion is quite distinct from the constant opening and closing of their shells (i.e., valves) or movement resulting from turbulent/high water flow.

From the surface up, there is the adhesive plaque and the thread, which is connected to the byssal retractor muscle hidden inside their shells. This muscle, controlled by a decentralized nervous system of paired ganglia, can contract and relax, thereby changing position of the animal relative to the substrate, as well as balance tension on the adhesive threads [17]. Perhaps an occasional tug induces mechanical strain on the thread and glue after deposition, helps to order the molecules, and creates a material well suited for living amongst the crushing waves of the intertidal zone.

### 1.3 Mussel Byssus and Mechanics

Mussels have created several strategies for mitigating mechanical forces from their surroundings. The byssal threads are often spread out in many directions, thereby aiding the ability to deal with forces from waves and turbulent waters [18,19,20]. These threads are made of a solid, non-porous material. The distal portion of threads, closest to the adhesive plaque on rocky substrates, is crystalline [18,21]. The proximal thread, nearest to the retractor muscles within the shell, is more elastic [18,21]. Generating such gradients of stiff to flexible, the threads may be tuned to dampen shocks. By contrast, the plaques have a microporous structure, potentially dissipating mechanical forces throughout the material [22]. Quite interestingly, this multi-component material changes in mechanical properties (e.g., tenacity) with seasons, wave action, food supply, pH, and temperature [23,24,25,26]. We wonder if forces, be they from the surroundings or the animals, are influencing the nature of the mussel’s attachment system. There has been some evidence suggesting that byssal threads can increase in material hardness with physical agitation or over time as the material ages [27,28]. Such motion, hydrated conditions, and the presence of dissolved oxygen in seawater could promote cross-linking based upon catechol chemistry. Perhaps internal forces could also play a role in curing mussel adhesives.

### 1.4 Animal Mechanics May Influence the Performance of Mimics

Our current, state of the art mimics of mussel adhesive proteins are often designed with a bottom-up approach. We start with small molecule insights, progress to larger molecules, such as plaques, and then examine bulk properties. When designing the next generation of biomimetic materials, we may wish to consider a more top-down approach from the animals’ perspective.

Examples of linking molecular with macroscopic design have been shown in the production of biomimetic silk [29,30,31]. Spiders tailor silk protein properties by specific processing with their spinnerets. Mechanical stresses induced by pulling on the silk helps to promote structural transitions from a semi-amorphous material to crystalline β-sheet and α-helical protein structures, giving silk the highly-prized properties of a strong, yet elastic, material [30,32].

It took years of effort to learn how such mechanical forces were essential for obtaining properties of real silk from mimics [30,31,32,33]. For example, silk proteins can be expressed in solution, isolated, and used to make materials, but the properties were always lacking when compared to genuine silk from spiders. Only when spinneret mechanical forces were incorporated could the desired, fully mimetic material properties be achieved.

Spider silk is composed of repeat glycine-, proline-, and alanine-rich peptide domains forming hierarchical structures for strength and elasticity [34]. Similarly, mussels have several mussel foot proteins (mfp) rich in glycine and uncharged residues [35]. Mussels might also use a pulling technique to modify the mechanical properties of their proteinaceous materials. This idea was proposed recently with regard to the mussel byssal threads [36]. These threads begin from secretory vesicles and end up being semi-crystalline structures. Mechanical forces from the animal may help organize the molecules into regular structures.

What about the adhesive plaques, themselves? In contrast to mussel and spider threads, the plaques are not crystalline. Rather, the adhesive has a foam-like structure [22]. Might mechanical forces help form the bulk material? We do know that formation of mussel plaques requires a large degree of cross-linking chemistry [1]. The DOPA residues in mussel foot proteins become oxidized [1,6,13,37]. Subsequent reactions with nucleophiles, such as reduced DOPA residues, amines, and thiols can bring about covalent cross-links to cure the material. However, this chemistry takes place within the solid plaque, or perhaps a precursor that is a foam or gel. In any case, mobility of reactive groups is low when compared to typical synthetic solution chemistry in which reagents are combined in solvents.

How do the reactive groups such as an electrophilic semiquinone and a nucleophilic, unoxidized DOPA find each other to couple within a solid material? We do know that such reactions are slow within this solid matrix given that radicals from iron-induced oxidation can be observed hours after the animals deposits this glue [13]. Most often in chemistry, radical species are so short-lived that they cannot be isolated and observed the way that they can for mussel adhesive. Given that these reactive groups appear to be at least somewhat trapped within a solid or semi-solid, we wonder if applied mechanical forces may aid curing.

**Figure 2:**
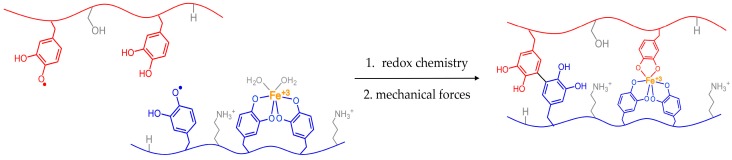
Possible scenario depicting how reactive species within a solid could be brought closer together via mechanical forces. Tugging from byssal retractor mussels could better align reactive groups for adhesive curing.

Figure 2 depicts a scenario in which reactive groups are physically separated from each other. Similar to spider silk, application of mechanical forces can shift orientation or placement of molecules with respect to each other. Bringing the electrophiles and nucleophiles closer together could then enable covalent coupling and curing. This chemistry could help to account for our observations of the animals tugging on their glue. If such interplay exists between curing chemistry and animal mechanics, we will then have an additional parameter to consider when processing mussel-mimicking synthetic polymers.

### 1.5 Testing the Influence of Forces from Mussels

After noticing consistent byssal ‘tugging’ by the animals, we became compelled to examine the potential effects of this behavior on material performance. We generated two sets of mussels and their adhesive: attached and detached. In the attached case, mussels deposited their glue onto surfaces and, after three days, the adhesive performance was measured. In the detached case, newly forged threads were immediately severed from the animal, resulting in samples that were connected to the animal for no more than 12 h.

**Figure 3:**
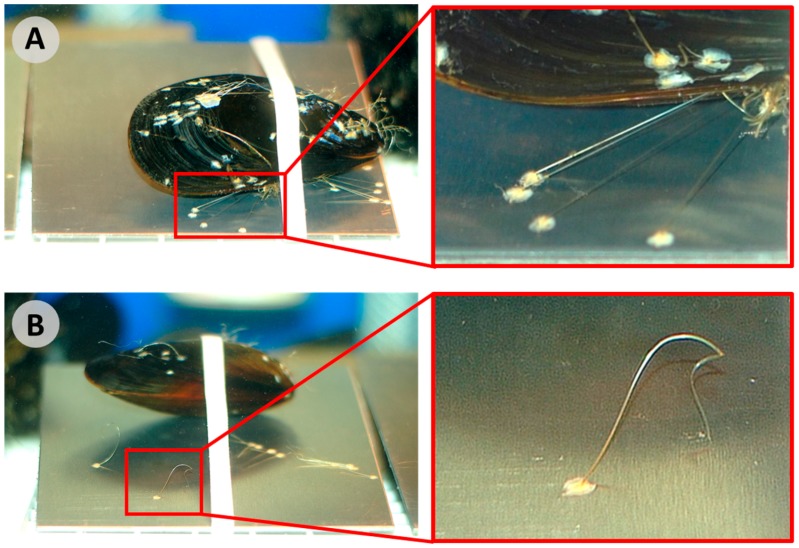
Experimental setup of marine mussels banded to aluminum substrates where: (**A**) Threads remain attached to the animal, taught and controlled by byssal retractor muscles; (**B**) Threads have been detached from the mussel at the closest point near their shell using a razor blade. The soft proximal portion of the threads can be seen where the threads start to curl and wave. The crystalline distal portions remain relatively straight.

Threads connecting animal and adhesive plaque were cut from the animal at the point providing the maximum thread length. These detached plaques were maintained in the same aquarium system as the animals to complete the three-day period (Figure 3). Animals were periodically checked and fresh adhesive was immediately detached from the animal (≤12 h) until completion of the three-day experiment. Adhesion performance was then assessed.

These experiments were carried out with both high surface energy 6061-T6 aluminum and low surface energy poly(methyl methacrylate) (PMMA, referred to as acrylic) substrate panels. Typical surface energy values fall around 41 and 169 mJ/m^2^ for acrylic and aluminum, respectively [38]. Exploiting low and high surface energy substrates gives insights on the nature in which these adhesives fail, which is further discussed in Section 3.1. In all, four datasets were created—attached on aluminum, detached on aluminum, attached on acrylic, and detached on acrylic. In the end, property differences between attached and detached plaques were found to be quite minimal, but there were observed differences in plaque areas on the two different surfaces.

## 2. Materials and Methods

### 2.1. Animal Handling

Live blue mussels (*Mytilus edulis*) were obtained from fishermen in Maine, USA, and shipped to our laboratory. The animals were maintained in an aquarium system that has been described previously [14]. Briefly, the system entails water at 4 °C, with a surge system for periodic turbulent flow to mimic periodic shore breaks along a coast, and artificial day/night cycles to represent Maine in February. Mussels used for this study were all of 50–70 mm in length. Animal feeding remained constant throughout the data collection period, which consisted of an enriched phytoplankton diet (Phyto-Feast, Reef Nutrition, Campbell, CA) every two days.

### 2.2. Adhesive Deposition

If mussels are placed onto a new surface, they tend to “walk” away, finding neighbors to aggregate with and stick together. Consequently, we placed the animals onto substrate sheets while held loosely in place with rubber bands. The substrates used here were 10 × 10 cm sheets of 6061-T6 aluminum (Farmers Copper Ltd., Texas City, TX, USA) and PMMA (United States Plastic Corp., Lima, OH, USA). Aluminum substrates were cleaned with detergent, ethanol, and acetone rinses followed by 3× rinses with deionized water. Acrylic substrates were prepared in the same manner, excluding the acetone wash. Each mussel/substrate/rubber band assembly was connected to a large plastic grate via zip ties in order to ensure exposure to consistent flow rates in a turbulent environment. Attached and detached samples were alternately arranged along the grate to minimize any deviations in flow rate along the sample set [24]. All mussels were oriented in the same manner in order to reduce variations in drag produced from their shell, with their long axis perpendicular to the upcurrent and the narrower region of valves facing into the current [20]. We did note that the aluminum substrates became slightly darker after three days of exposure in salt water.

### 2.3. Attached versus Detached Adhesive

Mussels were divided into four groups: attached plaques and detached plaques, each on aluminum or acrylic substrates. For the attached data, animals were placed atop substrates and they formed plaques and threads. Mussels were kept in the turbulent aquarium and adhesion measurements were made after three days. After years of working with these animals, we found that a period of three days is a sufficient amount of time for the mussels to anchor themselves securely to a given substrate. For the detached data, animals were placed onto the sheets of aluminum or acrylic. Once the adhesive was deposited, threads were cut at a point adjacent to the shell. The threads were cut as soon as they were visible or, at the longest, within 12 h of deposition. If further adhesive was produced later, similar cuts were made. These substrates with plaques were maintained in the aquarium, without the respective animals, for a total of three days. Thus, all adhesion data were collected three days after placing the animals within the tank. The attached plaques remained with the mussels for the whole three days. The detached plaques away from mussels for at least 2.5 days.

### 2.4. Adhesion Measurements

At the end of the experiment, all remaining threads were cut from the animal and immediately tested for adhesive strength using an Instron testing system (Instron 5544, Instron, Norwood, MA, USA). Details of pulling plaques until failure were reported previously [14]. Briefly, clamps surrounded the threads, were tightened, and pulled normal from the surface until failure. The mussel byssus tends to be fanned out into several or all directions from the animal (Figure 1). Adhesion testing can involve pulling each thread/plaque at different angles from the surface [39]. In practice, we tend to prefer using a 90° pull, normal from the surface, for all measurements [14,15]. Use of this approach allows for rapid collection of large data volumes in a consistent manner [14,15]. Such considerations are especially important in studies like this current one in which we have examined almost 1000 plaques. All plaque areas were measured using digital photography and ImageJ software [40].

### 2.5. Statistics

A total of 36 mussels were used in this experiment, resulting in 18 mussels used for each attached and detached group. Rather than running experiments on 18 mussels per group at one time, two data runs were carried out. Each data run contained nine mussels for collection of attached and nine mussels for collection of detached adhesive. Data from the two runs were then pooled. This approach helps to minimize variabilities, such as animal behavior within a given time period [15,26]. Error bars provided show 95% confidence intervals.

Each adhesive plaque was treated as an individual replicate. Pooled data included an average of all plaques, which we tend to refer to as ‘average of all’. We have also examined the average values for each mussel, and pooled those data separately, which we refer to as ‘average of the average’. There were no significant differences between these calculation methods, except in the magnitude of the error bars.

Statistics were calculated using SPSS software (IBM Corp., Armonk, NY, USA). An independent Student’s *t*-test examined significant differences within adhesion, force of removal, plaque area, and work of adhesion variables. The assumption of homogeny of variances were assessed using Laverne’s test, where the a priori α was set at a value of 0.5. Alternate hypotheses were accepted at *p*-values less than 0.05. Significance (*) is indicated in the figures. No statistical differences were found within acrylic variables. Conversely, the aluminum group found significant differences between attached and detached values within adhesion, force, and work of adhesion variables. The different areas of adhesive plaques deposited on acrylic and aluminum substrates were also statistically significant.

## 3. Results and Discussion

### 3.1. Adhesive Production and Failure Modes

For each dataset (attached aluminum, detached aluminum, attached acrylic, detached acrylic), 18 animals were used. Table 1 provides the pooled data for the plaques produced by the animals in each case. In all, 923 plaques were deposited. There are several ways in which mussel adhesive may fail under these conditions [14,39]. The plaque can be completely removed off the surface (“adhesive failure”), the plaque itself may tear apart (“cohesive failure”), the junction of plaque and thread might break (“thread–plaque failure”) or the thread may become severed (“thread breakage”). In practice, thread breakage is rare given that we cover the entire thread with the clamp providing the forces.

Speaking generally for all adhesives, high energy surfaces, such as metals or rocks, yield strong adhesion, whereas bonds are less robust on low-energy plastics. Translated to the conditions used here, mussel plaques are likely to exhibit significant degrees of cohesive failure on aluminum. Table 1 shows both the attached and detached plaques on aluminum failed cohesively just over half the time. Adhesive failure comprised the majority of remaining events. By contrast, weaker bonding to acrylic was manifested in a high percentage of adhesive failure, at 84% or more (Table 1). When comparing attached versus detached plaques on either surface, there were no major differences in distribution of failure modes. In at least this regard, the animals do not appear to be exerting any influence over the material properties.

### 3.2. Overall Adhesion

**Figure 4:**
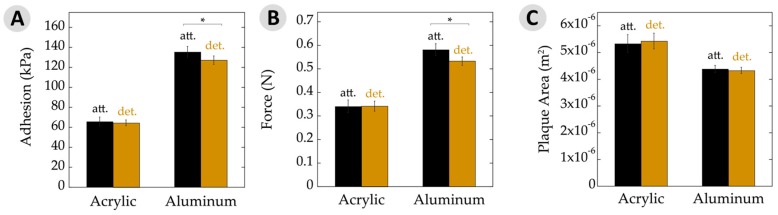
Adhesive properties. (**A**) Average adhesion of attached (att.) and detached (det.) plaques and threads on acrylic and aluminum substrates measured in kilopascals. Adhesion was calculated as a function of removal force divided by plaque area. Note overall increase in adhesion seen on a high surface energy substrate (aluminum) relative to a low energy surface (acrylic); (**B**) Force of byssal removal on acrylic and aluminum substrates measured in newtons; (**C**) Average plaque area deposited on acrylic and aluminum substrates. Note the change in plaque areas on acrylic versus aluminum. Asterisks (*) indicate statistically significant differences (*p* ≤ 0.05). All error bars show 95% confidence intervals.

For the low-energy acrylic substrate, Figure 4A shows the adhesion results. Performance of plaques that remained attached to the animals showed no significant difference. Adhesion is often expressed as the force of detachment divided by the overlap area. Hence, Figure 4B,C separate out these values. The force at failure, in Newtons, was very similar for attached and detached plaques on acrylic. Similarly, the plaque areas upon a given substrate remained quite constant between these two datasets.

The results slightly changed when looking at mussels on aluminum (Figure 4). The attached plaques showed a small, yet significant, increase in adhesion to the detached counterparts. Likewise, the maximum force at failure was very similar and the plaque areas were nearly identical for those attached versus detached. With aluminum being a high-energy surface and giving rise to generally high adhesion, potential differences might be seen more easily here versus with the acrylic surfaces. Nonetheless, the performance was very similar for adhesion, force at failure, and area.

There is often a direct relationship between adhesive strength and the substrate surface energy [38,41,42]. High-energy surfaces increase the spreadability of the adhesive due to enhanced adsorption and surface binding, yielding an overall stronger bond relative to a low-energy surface. This phenomenon was demonstrated by the changes to overall force and adhesion, where values increased by nearly 100% on aluminum versus acrylic. Interestingly, the adhesive area increased by ≈30% on low-energy acrylic compared to high-energy aluminum. There has been research investigating the relationship between the area of plaques and surface energy, but the reported results are somewhat contradictory [14,43,44,45,46]. In the case of adhesive plaques, the mfp-5, mfp-3f, and mfp-3s proteins are deposited along the adhesive–substrate interface [10,47]. The mpf-5 and mfp-3 proteins have the highest DOPA contents at 30% and 19%, respectively. Perhaps the mussel is able to control the spatial deposition and differentiate between proteins. They may then be able to maximize substrate binding via increasing mfp-5 and -3 at the interface of a low-energy substrate by upregulation of these proteins, using mechanical forces to increase the adhesive area, or simply the spreadability of the adhesive proteins behaves differently as the surface energy is changed.

Similarly, a larger plaque area would increase the overall protein as well as catechols in contact with the surface. These changes could thus aid animal binding to low-energy surfaces and explain the larger plaques observed versus upon high-energy aluminum. Placing such observations within the context of designing biomimetic adhesives may teach us two lessons. First, greater overlap area will enhance bonding to plastics. This idea is neither surprising nor unprecedented. More useful, however, may be the second prediction. Mussel mimicking polymers may exhibit higher adhesion between low-energy substrates when the catechol content is greater than those used to bind high-energy surfaces. In other words, polymers designed with a low catechol content make better adhesives on metals and other high-energy surfaces, whereas polymers with a higher catechol content may be used for bonding low-energy plastics. 

### 3.3. Work of Adhesion

**Figure 5:**
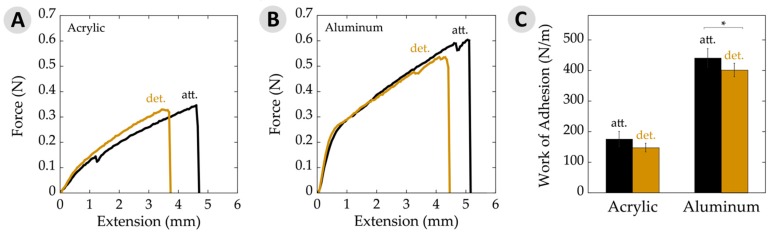
Work of adhesion data per unit area. (**A**) Typical force–extension curves on acrylic for attached (att.) and detached (det.) samples. Curves represent average plaque area, force, and extension values seen on acrylic; (**B**) Typical force–extension curves on aluminum substrates for attached and detached samples. Curves represent average plaque area, force, and extension values seen on aluminum; (**C**) Work of adhesion per unit area showing attached and detached values on acrylic and aluminum substrates. The asterisk (*) indicates a statistically significant difference (*p* ≤ 0.05). Error bars depict 95% confidence intervals.

Measuring the maximum load at failure of an adhesive material is a common way to express the relevant force. An alternative view on the strength of a bond is the energy required to rupture the joint. This work of adhesion is determined by integrating the area under force-versus-extension curves and factoring in overlap area [38,41,42]. Figure 5A,B shows such plots for the cases of adhesive failure on acrylic and aluminum for typical attached and detached plaques, which generally looked similar in shape. It is interesting to note that force–extension graphs from aluminum samples initiated with a steep slope within the first 1 mm of extension, followed by a more gradual slope until failure. This yield has been observed in mechanical tests of mussel adhesive threads, suggesting that the change in slope corresponds with transition of forces between several constituents and/or phases within the material [28,48]. Our testing system is designed to reduce factors from the threads as much as possible by covering almost the entire thread with the clamps. Nearly all of the observed forces are, thus, to deform the adhesive plaque. These plots provide what may be the first evidence that yielding phenomena could also be at play with the plaques.

The work of adhesion values for attached and detached plaques, both on acrylic and aluminum are depicted in Figure 5C. Here, we chose to present data averaged over all failure modes. Nearly all failure was adhesive in nature when on acrylic. Some differences may be found when separating out, for example, adhesive versus cohesive failure on aluminum [49]. However, the general trends remained the same. The work of adhesion on aluminum substrates showed a slight, yet significant, decrease with detachment relative to the attached adhesive. Similarly, adhesive on acrylic substrates decreased a little when moving from being attached to detached, but the potential changes here remained within the error limits. The observed differences here may be statistically significant, but are, nonetheless, small.

## 4. Conclusions

Our growing understanding of bioadhesives has given rise to an array of biomimetic materials and applications development. While we make such new systems, we are still teasing out the details contained within the parent systems under the seas. Data presented here help to examine the degree to which mussels influence the performance of their glue after deposition onto the substrate. On both a low- and high-energy surface, bonding was more or less equivalent, whether or not the animal had access to the adhesive. Measured plaque areas increased on a low- versus high-energy surface, suggesting that the animals have means to maximize bonding to a variety of surfaces. The observed tugging of mussels upon their byssus had very little influence on adhesion. These results indicate that future adhesive mimics may slightly benefit from mechanical pre-stressing when on high-energy surfaces. Mimics of mussel threads and spider silks, by contrast, are likely to improve properties with applied mechanical forces during processing. Perhaps the tugging we are seeing is the animals behaving in a manner akin to stretching when waking up in the morning.

## Figures and Tables

**Table 1:** Adhesion results of attached versus detached byssus on acrylic and aluminum.

	Acrylic		Aluminum	
	Detached	Attached	Detached	Attached
Number of plaques	231	134	314	244
Average adhesion (kPa)	65 ± 3	66 ± 4	127 ± 4	135 ± 5
Force of detachment (N)	0.34 ± 0.02	0.34 ± 0.02	0.53 ± 0.02	0.58 ± 0.03
Plaque area (×10^−6^ m^2^)	5.4 ± 0.3	5.3 ± 0.3	4.3 ± 0.1	4.4 ± 0.2
Work of adhesion (N/m)	148 ± 14	176 ± 25	401 ± 22	441 ± 30
Adhesive failure %	90.0	84.2	26.8	23.4
Cohesive failure %	4.8	7.5	57.3	58.6
Thread break failure %	3.5	3.0	10.8	8.6
Thread–plaque interface failure %	1.7	5.3	5.1	9.4
